# Supplementary material for: Assessing Microplastics and Nanoparticles in the Surface Seawater of Venice Lagoon—Part I: Methodology of Research
Source: Materials (Basel). 2024 Apr 11;17(8):1759. doi: 10.3390/ma17081759 (PMC11051501; doi:10.3390/ma17081759)
Supplement: Supplementary file 1 [file materials-17-01759-s001.zip › materials-2914700-supplementary.pdf]

materials

MDPI

Article

Assessing Microplastics and Nanoparticles in the Surface Seawater of Venice Lagoon—Part I: Methodology of Research

Teresa Cecchi [1], Davide Poletto [2,*], Andrei Constantin Berbecaru [3], Elfrida Mihaela Cârstea [4] and Maria Râpă [3,*]

[1] Chemistry Department, Istituto Technico Technologico, Via Montani 7, 63900 Fermo, Italy; cecchi.teresa@istitutomontani.edu.it
[2] Venice Lagoon Plastic Free, Castello 2641, 30122 Venice, Italy
[3] Faculty of Materials Science and Engineering, National University of Science and Technology Politehnica Bucharest, 060042 Bucharest, Romania; andrei.berbecaru@upb.ro
[4] National Institute of R&D for Optoelectronics INOE 2000, Atomistilor 409, 077125 Magurele, Romania; elfrida.carstea@inoe.ro
* Correspondence: d.poletto@plasticfreevenice.org (D.P.); maria.rapa@upb.ro (M.R.)

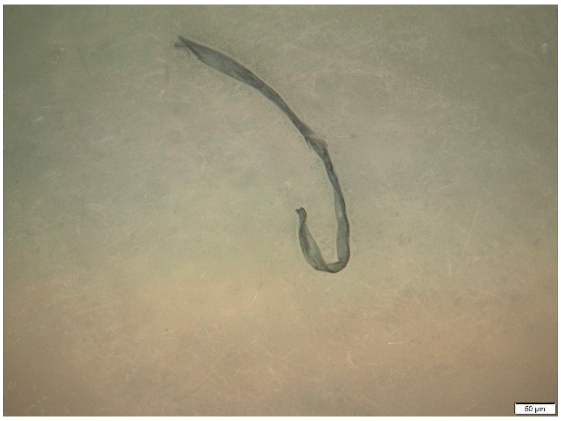

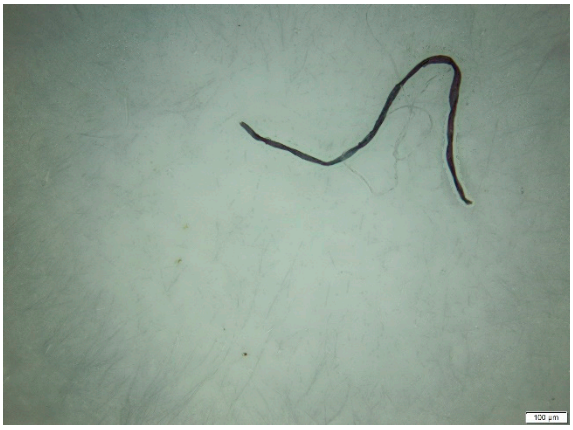

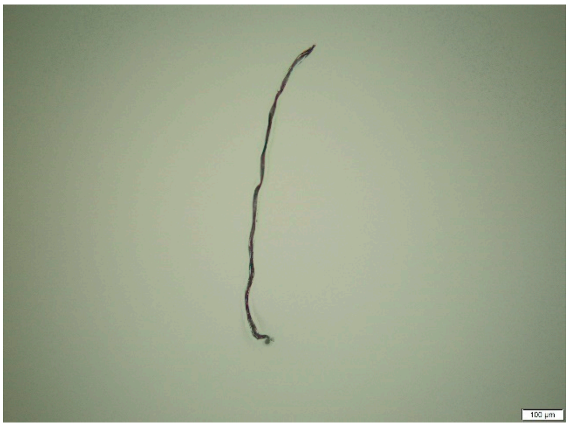

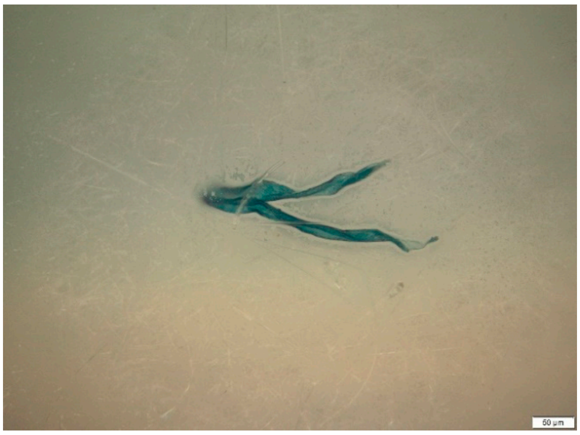

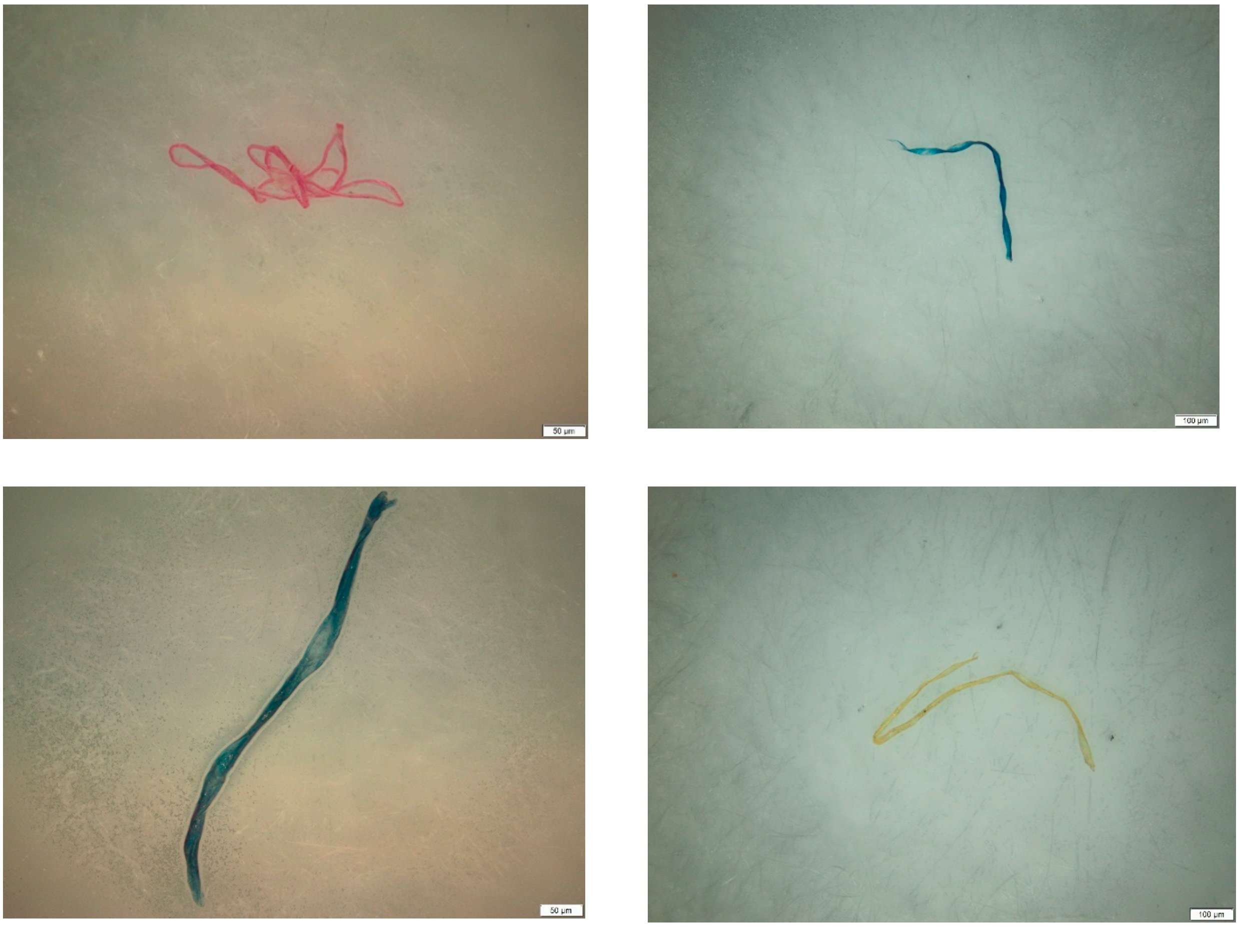

(a)

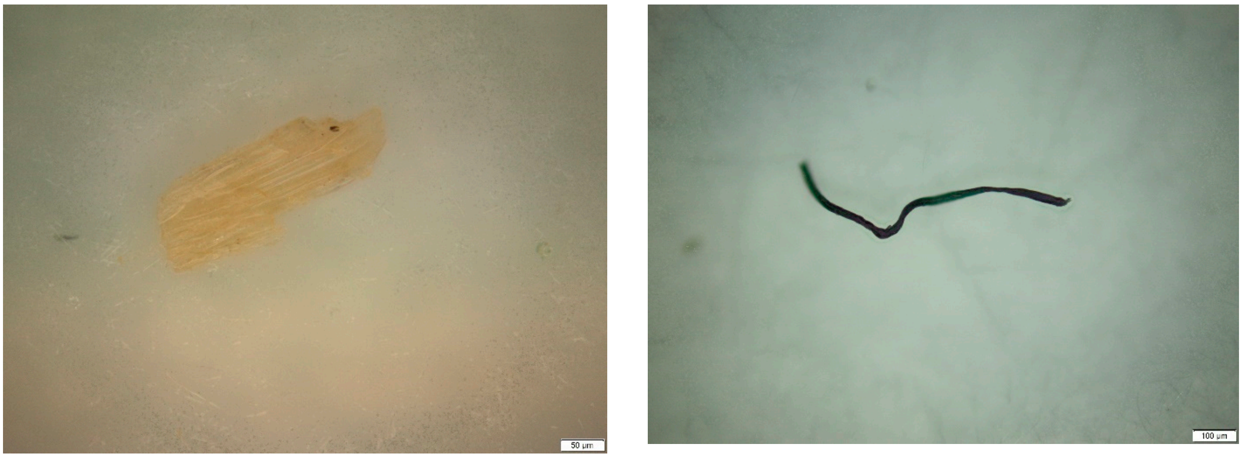

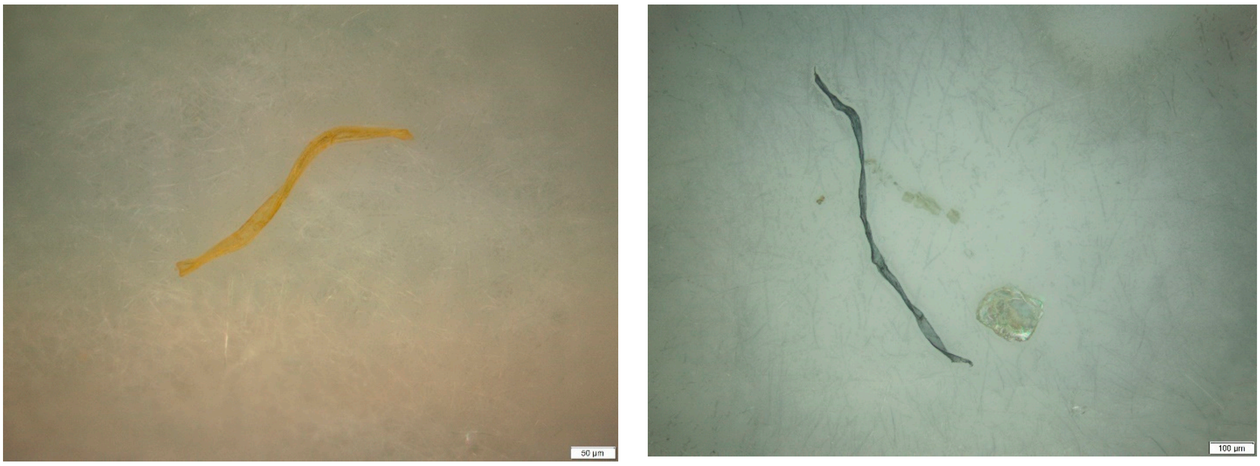

(b)

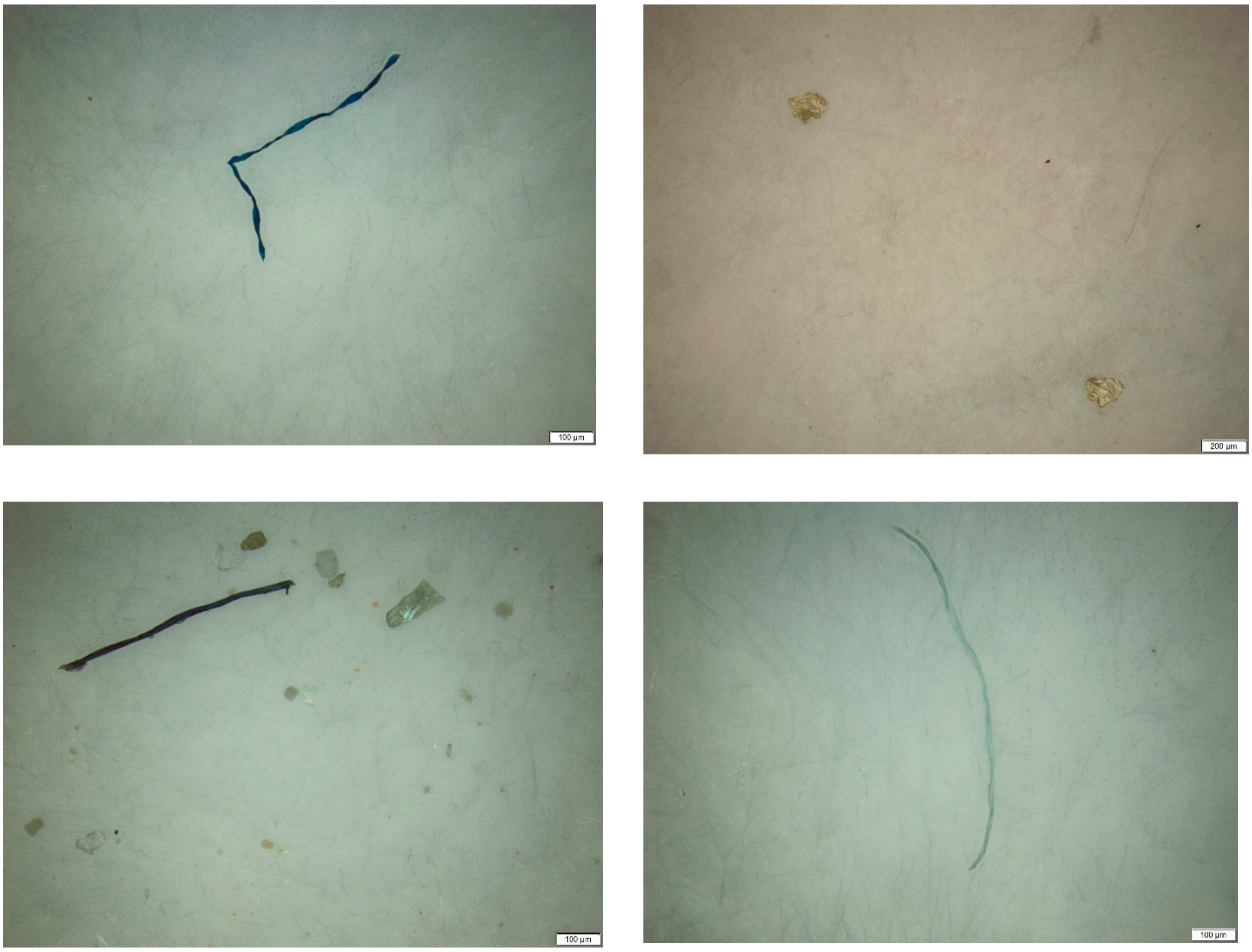

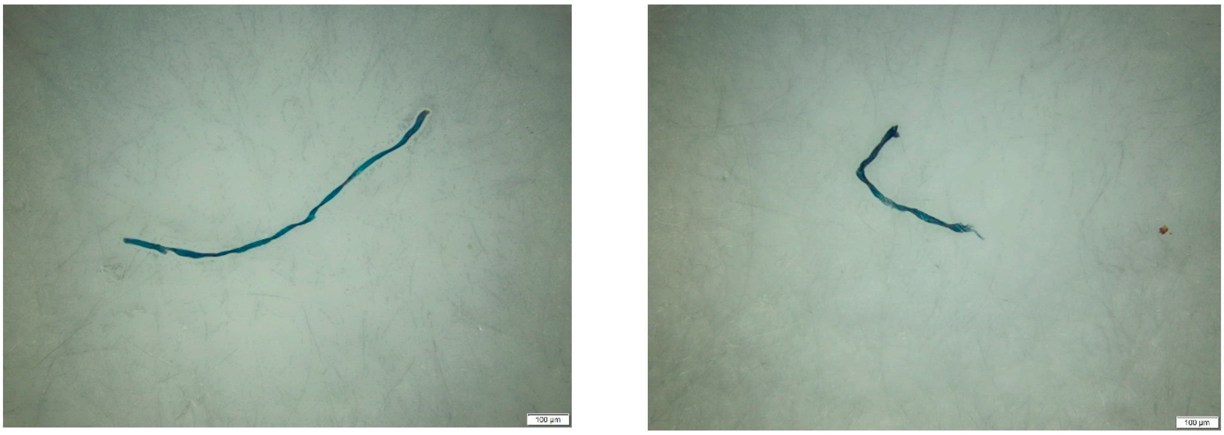

(c)

Figure S1: Some micrographs of MPs detected in seawater samples from Venice Lagoon: (a) Venice-Lido port inlet, (b) Grand Canal at the Rialto Bridge, and (c) Saint Marc basin.

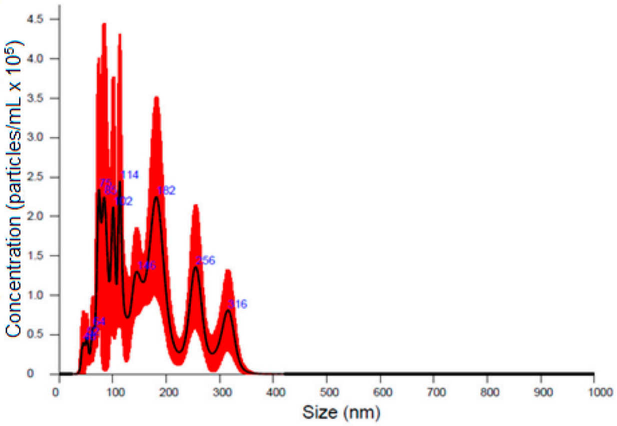

(a)

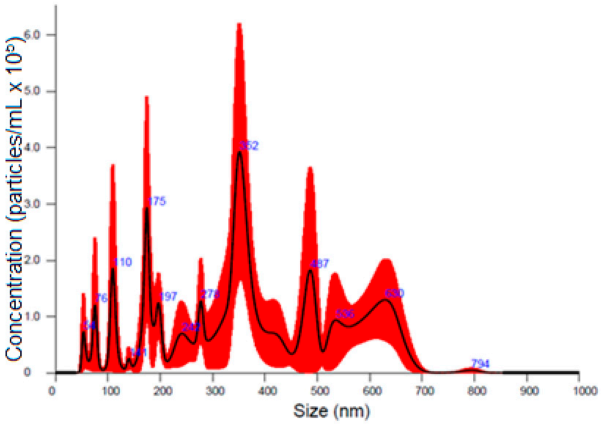

(b)

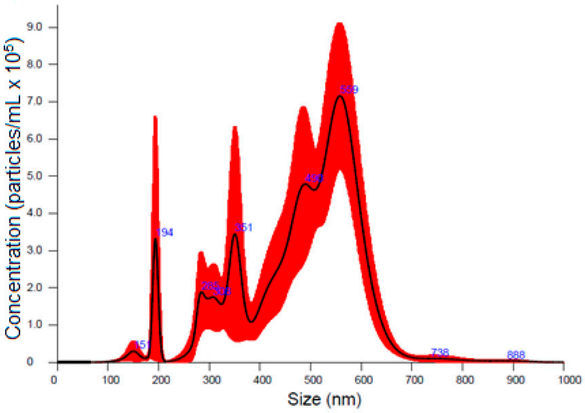

(c)

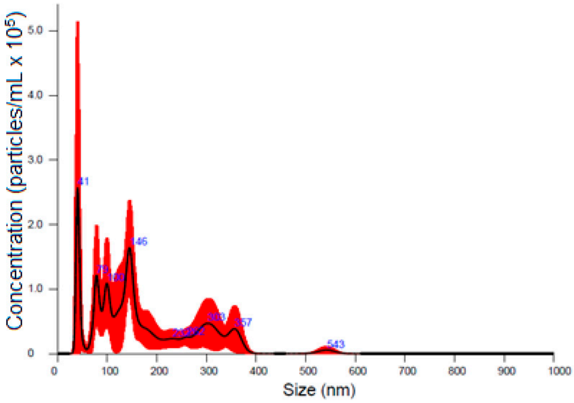

(d)

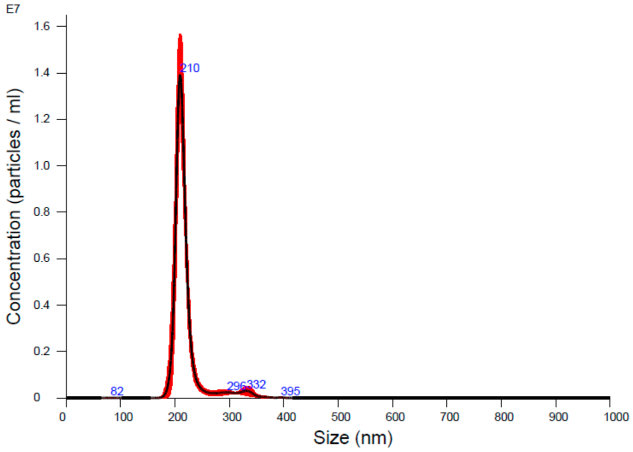

(e)

Figure S2: Averaged finite track length adjustment (FTLA) concentration/size for: (a) Venice- Lido port inlet, (b) Grand Canal at the Rialto Bridge, (c) Saint Marc basin, (d) Ultrapure water, and (e) Positive control.
